# Talar and Subtalar T1ρ Relaxation Times in Limbs with and without Chronic Ankle Instability

**DOI:** 10.1177/1947603521994626

**Published:** 2021-02-15

**Authors:** Kyeongtak Song, Brian Pietrosimone, Joshua N. Tennant, Daniel B. Nissman, Katherine M. Dederer, Chinmay Paranjape, Erik A. Wikstrom

**Affiliations:** 1 MOTION Science Institute, University of North Carolina, Chapel Hill, NC, USA; 2 Department of Orthopaedics, University of North Carolina, Chapel Hill, NC, USA; 3 Department of Radiology, University of North Carolina, Chapel Hill, NC, USA; 4 Panorama Orthopedics & Spine Center, Golden, CO, USA

**Keywords:** magnetic resonance, posttraumatic osteoarthritis, ankle sprain

## Abstract

**Objective:**

The primary aim was to determine differences in talocrural and subtalar joint (STJ) articular cartilage composition, using T1ρ magnetic resonance imaging (MRI) relaxation times, between limbs in individuals with unilateral chronic ankle instability (CAI) and compare with an uninjured control. Our secondary purpose was to determine the association between talocrural and STJ composition in limbs with and without CAI.

**Design:**

T1ρ MRI relaxation times were collected on 15 CAI (11 females, 21.13 ± 1.81 years, body mass index [BMI] = 23.96 ± 2.74 kg/m^2^) and 15 uninjured control individuals (11 females, 21.07 ± 2.55 years, BMI = 24.59 ± 3.44 kg/m^2^). Talocrural cartilage was segmented manually to identify the overall talar dome. The SJT cartilage was segmented manually to identify the anterior, medial, and posterior regions of interest consistent with STJ anatomical articulations. For each segmented area, a T1ρ relaxation time mean and variability value was calculated. Greater T1ρ relaxation times were interpreted as decreased proteoglycan content.

**Results:**

Individuals with CAI demonstrated a higher involved limb talocrural T1ρ mean and variability relative to their contralateral limb (*P* < 0.05) and the healthy control limb (*P* < 0.05). The CAI-involved limb also had a higher posterior STJ T1ρ mean relative to the healthy control limb (*P* < 0.05). In healthy controls (*P* < 0.05), but not the CAI-involved or contralateral limbs (p>0.05), talocrural and posterior STJ composition measures were positively associated.

**Conclusions:**

Individuals with CAI have lower proteoglycan content in both the talocrural and posterior STJ in their involved limbs relative to the contralateral and a healthy control limb. Cartilage composition findings may be consistent with the early development of posttraumatic osteoarthritis.

Chronic ankle instability (CAI) is a common sequela following acute lateral ankle sprains^1-3^ and both lateral ankle sprains and CAI increase the risk of posttraumatic osteoarthritis (PTOA) development at the ankle.^4,5^ The onset of ankle PTOA (i.e., compositional changes) may begin within the first several years following injury with CAI onset.^6,7^ Thus developing our understanding of early compositional changes may facilitate therapeutic development and slow PTOA progression. The existing ankle literature has focused almost exclusively on a T2-mapping compositional magnetic resonance (MR) scan of the talocrural joint (i.e., talar dome) in a CAI-involved limb relative to a healthy control limb.^8-10^ However, the earliest compositional change of PTOA is a loss of proteoglycan content which is associated with early PTOA development and can be captured using a T1ρ compositional MR scan.^11^ Those with CAI have been shown to have a higher T1ρ mean (i.e., lower proteoglycan content) as well as higher T1ρ variability across the talar dome of the involved limb relative to a healthy control limb.^12^ However, no between-limb investigation (i.e., CAI-involved and -contralateral limb) has quantified proteoglycan content in those with unilateral CAI and compared these values with a healthy control limb, despite asymmetrical movement patterns having been observed in those with CAI.^13^

The subtalar joint (STJ) ligaments are often concurrently damaged during a lateral ankle sprain,^14^ and evidence indicates that STJ instability increases with lateral ankle ligament sectioning^15^ and laxity in those with a history of a lateral ankle sprain.^16^ Thus, STJ instability is likely present in those with CAI. If untreated, STJ instability impairs mobility and could contribute to abnormal biomechanics and/or talocrural degeneration in those with CAI. Given the role that the STJ plays in shock absorption,^17^ impaired STJ mobility may also contribute to STJ degeneration over time.^18^ However, only a single investigation has quantified STJ cartilage composition, via T2-mapping, in those with CAI.^19^ In healthy individuals, the STJ and shank are thought to be kinematically coupled as a result of ankle joint complex anatomy.^20^ Thus CAI appears to disrupt the normal shank-rearfoot coupling seen in healthy individuals, likely because of STJ instability (i.e., ligamentous laxity).^21-23^ As a result of a kinematic decoupling in those with CAI, the talocrural and STJ likely respond to mechanical loads differently. If true, cartilage composition between the talocrural and STJ joints could also differ and result in accelerated proteoglycan content changes in one joint relative to the other. To address this knowledge gap, a concurrent assessment of talocrural and STJ proteoglycan content in CAI and healthy cohorts is needed.

Therefore, the primary purpose of this investigation was to determine differences in talocrural and STJ proteoglycan content among a CAI-involved, a CAI-contralateral, and a healthy control limb. Our secondary purpose was to determine the association between talocrural and STJ proteoglycan content in a CAI-involved and a healthy control limb. To achieve these objectives, we will evaluate T1ρ relaxation time means and variability in the talocrural and STJ. We hypothesize that both the CAI-involved and -contralateral limbs would have higher T1ρ relaxation times (i.e., lower proteoglycan content) and greater T1ρ variability relative to the healthy control limb. We also hypothesized that T1ρ relaxation time means and T1ρ variability of the talocrural and STJ would associate in a healthy control limb but not in the CAI-involved limb.

## Methods

### Design and Participants

A cross-sectional design was used to address the stated research questions and we used the STROBE (Strengthening the Reporting of Observational studies in Epidemiology) cross-sectional reporting guidelines.^24^ All participants were between 18 and 35 years of age and free from acute lower extremity and head injuries for at least 3 months prior to testing. Anyone with equilibrium disorders or symptoms from musculoskeletal or head injuries sustained at any time was excluded. Controls demonstrated an Identification of Functional Ankle Instability (IdFAI) score of <11, a Foot & Ankle Ability Measure Activities of Daily Living subscale (FAAM-ADL) score of ≥98%, and a Foot & Ankle Ability Measure Sport subscale (FAAM-S) score of ≥98%. Individuals with CAI had a history of at least 1 lateral ankle sprain and at least 2 episodes of giving way within the past 6 months; an IdFAI > 11, a FAAM-ADL < 90%, and a FAAM-S < 80% per the International Ankle Consortium guidelines.^25^ International Ankle Consortium recommendations for exclusion criteria were also followed.^25^ Rearfoot alignment was not quantified in the sample. Three participants presented with bilateral CAI and were excluded from the CAI between limb comparison. For the remaining analyses, the limb with more ankle sprains and worse self-reported function was used as the involved limb. Written informed consent was provided prior to participation and all procedures were approved by the university’s biomedical ethics review board. Published STJ T2 values indicate a large bias corrected effect size of 1.36 between CAI and control groups.^19^ However, we planned for a more conservative effect (0.54) in line with talocrural T2 values between CAI and control groups.^10^ Coupled with an alpha level of 0.05 and 1 − β of 0.80, the total needed sample size was 30 was needed to detect differences.

### Magnetic Resonance Image Acquisition

A Siemens Magnetom TIM Prisma 3T scanner and an 8-channel large flex coil (516 mm × 224 mm, Siemens, Munich, Germany) acquired anatomical (PD space) and T1ρ MR images. MR was performed with the foot/ankle complex in neutral (90° to the shank) and the participant supine after a 30-minute unloading (i.e., nonweightbearing) period prior to the scan.^12^ A T1ρ prepared 3-dimensional fast low angle shot (FLASH) sequence with a spin lock power at 500 Hz and a bandwidth of 350 Hz/Px was used.^12^ Spin lock durations included 40, 30, 20, 10, and 0 ms. Voxel size was 0.8 mm × 0.4 mm × 3 mm (field of view = 288 mm, slice thickness = 3.0 mm, repetition time [TR] = 9.2 ms, 160 × 320 matrix, gap = 0 mm, flip angle = 10°, echo train duration time = 443 ms, phase encode direction of anterior/posterior). T1ρ acquisition time for each limb was approximately 12 minutes.^12^

### T1ρ Relaxation Time Quantification

A five image series created with a custom MatLab program (MatLab R2016b [9.1.0] MathWorks, Natick, MA, USA) calculated Voxel by voxel T1ρ relaxation times using the following equation: S(TSL) = S_0_ exp(−TSL/T1ρ).^12^ The S corresponds to signal, TSL is the length of the spin-lock time, S_0_ is the signal intensity when TSL equals 0, and T1ρ is the T1 relaxation time in the rotating frame. The program used a 2-parameter fit and excluded low-signal regions that fit poorly with the model. The talocrural and STJ were manually segmented using ITK-SNAP version 3.2 software.^12^ The talocrural and STJ cartilage for each limb was segmented so that T1ρ mean and variability (i.e., standard deviation of the T1ρ values across the cartilage) were calculated.^12^ For this investigation, a laminar analysis of the talar dome was not conducted because previous research demonstrated that those with CAI had consistently worse composition in both the superficial and deep layer relative to uninjured controls.^10^ The STJ segmentation created an anterior, medial, and posterior region of interest consistent with STJ anatomical articulations.^26^ Overall STJ values were not calculated as they are heavily influenced by the size of the posterior STJ articulation. For this investigation, STJ cartilage was defined as the full thickness of the talocalcaneal cartilage because of concerns about talocalcaneal thickness (range: 0.55-1.00mm) and having enough voxel rows per cartilage layer if it were to be divided.^26-28^

Following segmentation, the primary and secondary T1ρ outcomes were extracted for each joint and used for further analysis. The T1ρ relaxation time mean and variability for the overall talocrural and posterior STJ articulation served as the primary outcome measures. Greater T1ρ relaxation times were interpreted as greater cartilage degeneration.^11,12^ T1ρ variability was defined as the standard deviation of T1ρ relaxation times across all pixels of an articulation and higher T1ρ variability was interpreted as a more diffuse distribution of degenerative changes within the cartilage of interest.^12^ Secondary measures included T1ρ mean and variability for the anterior and medial STJ articulations. The CAI-involved and -contralateral limbs as well as the right and left controls limbs were not registered (i.e., aligned) because we focused on an entire joint surface as opposed to a specific region of interest. Total cartilage volume of the talocrural and STJ articulation was calculated to account for potential differences in ankle size and/or usable slices. Additionally, a fellowship trained foot and ankle surgeon and 2 orthopaedic residents evaluated the anatomical sequences for degenerative changes. While a standardized scoring regimen was not used, differences in findings were discussed until consensus was reached.

### Statistical Analysis

Participant demographics were compared between groups using independent *t* tests. To quantify the relationship between morphology and the primary T1ρ outcome, several analyses were conducted. Talocrural and STJ cartilage volume was compared among the limbs using separate 1-way analyses of variance. Associations between talocrural and STJ cartilage volume and the primary outcome measures were established, for each limb group independently, via Pearson correlations. Finally, the frequency of degenerative observations was compared between the groups using a chi-square analysis.

A preliminary analysis compared the dominant and nondominant limbs of the control group and noted no differences in the primary outcome measures (*P* ≥ 0.209). Thus, a between-limb average was calculated for the primary and secondary variables for the healthy control limb. The primary variables (i.e., overall talocrural and posterior STJ T1ρ relaxation time means and variability) were then compared between the CAI limbs (i.e., involved and contralateral) using paired *t* tests and between each CAI limb and the healthy control limb using independent *t* tests. Secondary STJ variables (i.e., anterior and medial STJ T1ρ relaxation time means and variability) were submitted to identical analyses. The CAI-contralateral limb group consisted of contralateral limbs (*n* = 12) that did not meet our a priori definition of CAI. Bias corrected Hedge’s *g* effect sizes with their corresponding 95% confidence intervals (95% CIs) were also calculated for all between-limb comparisons.

Associations between the overall talocrural and posterior STJ T1ρ relaxation time means and variability (primary variables) within the CAI-involved, CAI-contralateral, and healthy control limb data were determined using separate Pearson correlations. Correlations were interpreted as weak with a correlation coefficient from 0.01 to 0.39, moderate from 0.40 to 0.69, and strong from 0.70 to 1.00.^29^ SPSS Version 21.0 (IBM Corp, Armonk, NY, USA) was used and an alpha level of 0.05 was used to determine statistical significance for all analyses.

## Results

Fifteen uninjured controls and 15 individuals with CAI participated. Demographics and injury history means and standard deviations can be seen in **Table 1**. Demographics did not differ among the groups (*P* > 0.05) but injury history characteristics differed among the CAI-involved, CAI-contralateral, and control limbs (*P* < 0.05).

**Table 1.** Means, Standard Deviations, and *P* Values for Demographic and Injury History Variables.

Demographic Variables	CAI Group (*n* = 15)		Control Group (*n* = 15)
Sex	4 males; 11 females		4 males; 11 females
Age (years)	21.13 ± 1.81		21.07 ± 2.55
Height (cm)	166.62 ± 8.08		167.18 ± 7.73
Weight (kg)	66.50 ± 8.27		69.21 ± 13.60
Injury History Variables	CAI-Involved (*n* = 15)	CAI-Contralateral (*n* = 12)	Control Limb (*n* = 15)
Number of ankle sprains	4.00 ± 2.07* †	1.25 ± 1.36*	No previous sprains
Episodes of giving way within the past 6 months	6.87 ± 5.36* †	No giving way episodes	No giving way episodes
IdFAI scores	22.67 ± 2.82* †	9.75 ± 8.17*	0.13 ± 0.52
FAAM (%)	86.19 ± 9.77* †	98.22 ± 3.07	100.00 ± 0.00
FAAM-Sport (%)	68.33 ± 21.87* †	91.67 ± 13.41	100.00 ± 0.00

CAI = chronic ankle instability; IdFAI = Identification of Functional Ankle Instability; FAAM = Foot & Ankle Ability Measure.

* Indicates a significant difference from the control limb (*P* < 0.05).

† Indicates a significant difference from the CAI-contralateral limb (*P* < 0.05).

No differences in cartilage morphology were observed among the CAI and control groups. Overall talar cartilage volume (CAI-involved, 1581.92 ± 335.74 mm^3^; CAI-contralateral, 1466.4 ± 245.54 mm^3^; control, 1446.37 ± 294.31 mm^3^; *P* = 0.403) and posterior STJ cartilage volume (CAI-involved, 1787.67 ± 379 mm^3^; CAI-contralateral, 1772.93 ± 338.2 mm^3^; control, 1881.12 ± 451.6 mm^3^; *P* = 0.718) did not differ between the groups. Talocral and STJ cartilage volume of the CAI-involved, CAI-uninvolved, and control limbs demonstrated nonsignificant (*P* > 0.05) and weak associations (range: −0.300 to 0.093) for all primary outcome measures. Similarly, the frequency of visible morphologic changes was not different between groups (*P* = 0.256). A total of 4 (27%) of 15 uninjured controls and 7 (47%) of 15 individuals with CAI were observed to have degenerative changes. Individuals within the control group had the following morphology findings: marrow edema (*n* = 1), a subchondral cyst (*n* = 1), a local osteochondral defect (*n* = 1), and talar osteophytes (*n* = 1). Individuals with CAI were found to have the following morphologic changes: a subchondral cyst (*n* = 3), talar osteophytes (*n* = 2), chondral delamination (*n* = 1), and anterolateral cartilage thinning (*n* = 1). The changes are thought to be the result of traumatic chondral shear forces.

**Figure 1.**
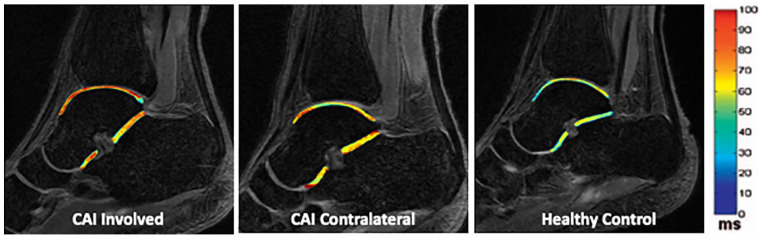
Exemplar heat map of the talocrural and subtalar joint T1ρ means in the chronic ankle instability (CAI) involved limb (left), CAI contralateral limb (center), and the average control limb (right). Warmer colors represent higher T1ρ values and lower proteoglycan content.

The CAI-involved limb demonstrated a higher talocrural T1ρ relaxation time mean (i.e., less proteoglycan content) (*P* ≤ 0.032) and more T1ρ variability (*P* ≤ 0.002) relative to the CAI-contralateral limb and the healthy control limb (**Table 2**). The CAI-involved limb also demonstrated a higher posterior STJ mean T1ρ relaxation time (*P* = 0.034) relative to the healthy control limb (**Table 2**). These results were associated with large effect sizes with 95% CIs that did not cross zero. The combined results can be visualized in **Figure 1**, showing an exemplar heat map of the T1ρ means at both the talar and STJ joints between the CAI-involved, CAI-uninvolved, and healthy control limbs. The CAI-contralateral limb’s posterior STJ mean T1ρ relaxation time did not differ from the healthy control limb (*P* = 0.055) but were associated with a 95% CI that did not cross zero. Among the secondary STJ variables, only the medial STJ T1ρ relaxation time variability differed between the CAI-involved and the healthy control group (*P* = 0.043). This difference was associated with a large effect size and a 95% CI that did not cross zero. No other differences were noted among the primary or secondary variables (**Table 2**).

**Table 2.** Means, Standard Deviations, Effect Sizes, and 95% Confidence Intervals (95% CIs) for Primary and Secondary T1ρ Variables (ms).

	CAI-Involved (*n* = 15)	CAI-Contralateral (*n* = 12)	Control Limb (*n* = 15)	CAI-Involved – Control Effect Size (95% CI)	CAI-Involved – CAI Contralateral Effect Size (95% CI)	CAI-Contralateral – Control Effect Size (95% CI)
Primary talocrural variables						
T1ρ mean	65.97 ± 10.46* †	55.69 ± 12.47	56.31 ± 6.16	1.09 (0.33, 1.86)	0.88 (0.08, 1.67)	−0.06 (−0.82, 0.70)
T1ρ variability	32.78 ± 4.06* †	26.07 ± 4.93	27.23 ± 3.67	1.40 (0.60, 2.19)	1.46 (0.61, 2.31)	−0.26 (−1.03, 0.50)
Primary subtalar variables						
Posterior T1ρ mean	65.05 ± 4.76*	64.53 ± 4.39‡	60.71 ± 4.40	0.92 (0.17, 1.67)	0.10 (−0.66, 0.86)	0.80 (0.01, 1.59)
Posterior T1ρ variability	27.33 ± 5.23	27.08 ± 5.58	25.14 ± 5.33	0.40 (−0.32, 1.13)	0.05 (−0.71, 0.80)	0.35 (−0.42, 1.11)
Secondary subtalar variables						
Anterior T1ρ mean	66.98 ± 13.21	65.71 ± 13.38	65.90 ± 11.45	0.09 (−0.63, 0.80)	0.09 (−0.67, 0.85)	−0.01 (−0.77, 0.74)
Medial T1ρ mean	69.60 ± 7.71	67.25 ± 7.84	64.62 ± 7.84	0.62 (−0.11, 1.36)	0.29 (−0.47, 1.06)	0.33 (−0.44, 1.09)
Anterior T1ρ variability	35.62 ± 4.87	33.64 ± 5.46	31.51 ± 8.27	0.63 (−0.11, 1.36)	0.38 (−0.38, 1.15)	0.21 (−0.55, 0.97)
Medial T1ρ variability	32.46 ± 4.58*	30.07 ± 7.49	28.71 ± 5.10	0.75 (0.01, 1.49)	0.37 (−0.39, 1.14)	0.32 (−0.45, 1.08)

CAI = chronic ankle instability.

* Indicates a significant difference from the control limb (*P* < 0.05).

† Indicates a significant difference from the CAI-contralateral limb (*P* < 0.05).

‡ Indicates a statistical trend from the control limb (*P* = 0.055) and a 95% CI that does not cross zero.

**Figure 2.**
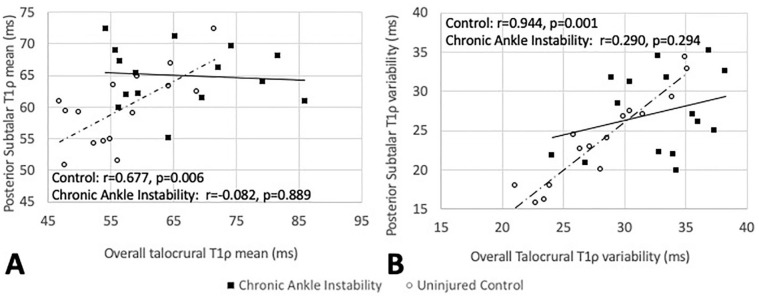
Association between the posterior subtalar joint and overall talar dome cartilage T1ρ means (**A**) and variability (**B**) in uninjured controls (open circles and dashed line) and the chronic ankle instability involved limb (closed squares and solid line).

A moderate positive association was noted between greater posterior STJ T1ρ mean and greater overall talocrural T1ρ mean relaxation times (*r* = 0.677, *P* = 0.006) in healthy individuals (**Fig. 2A**). However, in the CAI-involved limb (*r* = −0.082, *P* = 0.889) and CAI-contralateral limb (*r* = 0.300, *P* = 0.343), weak associations that were not statistically significant were observed between the overall talocrural and posterior STJ T1ρ relaxation time means (**Fig. 2A**). Similarly, a strong positive association between the posterior STJ T1ρ variability and the overall talocrural T1ρ variability was noted (*r* = 0.944, *P* < 0.001) in healthy controls (**Fig. 2B**). In the CAI-involved limb (*r* = 0.290, *P* = 0.294) and CAI-contralateral limb (*r* = −0.157, *P* = 0.626), weak nonsignificant relationships were noted between the overall talocrural and posterior STJ T1ρ variability (**Fig. 2B**).

## Discussion

Our primary findings support the notion that CAI is associated with deleterious changes in talocrural and posterior STJ cartilage composition compared with healthy control limbs. Our findings also suggest that the CAI-contralateral limb may have deleterious changes in the posterior STJ but not talocrural cartilage composition relative to a healthy control limb. Last, the results demonstrate that moderate to strong associations were noted between talocrural and STJ composition values in the healthy control limbs while weak associations were noted between the compositional values in the CAI-involved and CAI-uninvolved limb. These results support our a priori hypotheses and are consistent with the existing literature. This is the first study to demonstrate deleterious changes in both talocrural and posterior STJ cartilage composition within the same cohort of CAI participants. These data are important as they highlight the need for an improved understanding of and interventions for lateral ankle sprain and CAI sequelae at the STJ.

### T1ρ Mean

Trauma to cartilage, including trauma during a lateral ankle sprain, results in the disruption of the cartilaginous matrix.^7,30,31^ This disruption is initiated by a reduction in proteoglycans, a primary structural element of the extracellular matrix, and can occur early post injury.^31,32^ Slowing PTOA progression is dependent on improving our understanding of these early deleterious changes. The T1ρ MR technique is sensitive to changes in proteoglycan content and higher T1ρ relaxation times are associated with lower proteoglycan content.^33,34^ The higher T1ρ means observed in the CAI-involved limb are consistent with the existing literature at both the talocrural and STJ.^8-10,19^ Further, the increased CAI-involved T1ρ relaxation time means at both the talocrural and STJ were noted despite comparable cartilage volumes at both joints among the CAI-involved, CAI-contralateral, and the healthy control limb. Specific to the STJ, our findings were consistent with the literature.^19^ While the exact mechanism responsible for the differences noted in individual CAI patients will vary, the existing literature highlights trauma to the cartilage during the index injury and altered joint loading over time as 2 common pathways.^4^

Differences in STJ cartilage composition between a CAI-involved and control limb are consistent across studies despite meaningful methodological differences. For example, STJ compositional differences have been noted in CAI patients that were and were not (present study) seeking medical care.^19^ Additionally, CAI-involved to control limb differences have been detected regardless of whether the posterior STJ articulation was subdivided into regions or layers. Previous research divided the posterior STJ into regions based on how the STJ aligned with the talar dome in the transverse plan.^19^ We chose to examine the posterior STJ as a single entity because previous research found compositional differences in all divisions and layers of the posterior STJ cartilage in CAI patients.^19^ We therefore examined the full thickness of the talocalcaneal cartilage^26^ because STJ cartilage is only 0.55 to 1.00 mm thick^27^ and we wanted to maximize the amount of available voxels that could be used to draw conclusions. We also segmented all STJ articulations using all available MR slices, which has not been done previously, to ensure the most comprehensive examination of STJ T1ρ relaxation time means and variability.

To our knowledge, this is the first investigation that has quantified cartilage composition of the talocrural or STJ in a CAI-contralateral limb. While this makes the CAI-contralateral results difficult to contextualize, our results seem to be articulation specific. For example, the CAI-contralateral talocrural T1ρ mean more closely resembles the control limb than the CAI-involved limb (**Table 1**). However, the contralateral posterior STJ mean is more comparable to the CAI-involved mean and trends toward differing from the healthy control limb. The reason for this potential articulation specific difference remains unknown but may be due to asymmetrical movement patterns developed post lateral ankle sprain,^13^ greater structural damage (e.g., bruising) at the talocrural joint during the injurious event, and/or preinjury profiles (e.g., biochemical, biomechanical) present in the STJ but not in the talocrural cartilage of those that go on to sustain a lateral ankle sprain and/or develop CAI. For example, there is an association between baseline measures of cartilage synthesis and degradation and a subsequent likelihood of anterior cruciate ligament injury.^35^ Further research is warranted to examine how these proposed factors could influence prospective talocrural and STJ cartilage composition changes in both limbs following acute lateral ankle sprains. Additionally, the standard deviation around the talocrural mean was higher in the CAI-uninvolved limb relative to the CAI-involved limb. This is likely due to the fact that the CAI-uninvolved limb was not required to be injury free and the standard deviation represents the variance among the ankles that made up the CAI-uninvolved limb mean. This does not imply a homogeneous injury history among the CAI-involved limbs but rather the established minimum (i.e., the inclusion criteria) may have reduced the variance among the group.

### T1ρ Variability

Talocrural T1ρ variability is elevated in the CAI-involved limb compared with a healthy control limb as previously reported.^12^ However, neither the CAI-contralateral talocrural articulation nor the STJ articulations differed from the healthy control limb. Unfortunately, a lack of published variability data limits our ability to hypothesize about the mechanisms behind our observations. However, when coupled with the T1ρ mean data (i.e., early deleterious changes in cartilage composition at both the talocrural and STJs), it appears that posterior STJ compositional declines are more uniform in nature. Further research is needed to test this hypothesis and determine if impact trauma and its associated sequela (e.g., bone bruising) or prolonged alterations in joint loading or both play a role in the uniformity of compositional changes (i.e., spatial distribution of variance) at both the talocrural and STJ using compositional variability (e.g., T1ρ variability) or another tool such as texture analysis.^36^

### Talocrural and Subtalar Joint Coupling

Multiple authors have highlighted the links between the talocrural and STJ.^14-16,37^ Yet, this is the first investigation to examine cartilage composition associations between the talar dome and posterior STJ articulation. Our most important finding was that the posterior STJ T1ρ relaxation time mean and variability demonstrated moderate to strong positive associations with the T1ρ mean and variability of the talar dome in a healthy control limb (**Fig. 2A** and **B**). However, weak and nonsignificant associations between these same articulations were noted in the CAI-involved and -uninvolved limbs. This result is likely due to a combination of the heterogeneous nature of the initial lateral ankle sprain (e.g., severity, comorbidities, treatment) and the heterogeneity of the residual impairments (e.g., balance, biomechanics, arthrokinematic restrictions) that are present in CAI. For example, in those with CAI, the biomechanical shank-rearfoot couple is disrupted relative to uninjured controls.^21-23^ Cumulatively, these results suggest that the coupled response to loading between the talocrural and STJ is disrupted in those with CAI, subsequently leading to different rates and patterns of deleterious changes within the talocrural and posterior STJ cartilage.

### Limitations

This study is the first to quantify talocrural and STJ composition on a CAI-involved, CAI-contralateral, and a healthy control limb but there are some limitations that can inform future research. While the current study provides novel information regarding STJ compositional differences between limbs with and without CAI, our results are from a small sample size of recreational active individuals. Thus, the results may not generalize to elite populations and/or those actively seeking medical care. We did not conduct a laminar analysis or correct for a potential magic angle effect at either the talocrural or STJ cartilage which should be done in future investigations to provide better and higher fidelity insight into the etiology of proteoglycan content depletion due to compression and/or shear forces. Similarly, future investigations should consider using a 3-parameter fit to improve T1ρ relaxation times signal to noise ratios and the use of an MRI trained radiologist to gain further insights into the etiology of chondral wear (i.e., chondral lesions with secondary cartilage loss or traumatic chondral shear). Our CAI-contralateral limb was required to be free from CAI but not required to be free from a previous lateral ankle sprain. As a result, the CAI-contralateral limb data may be driven by the initial ankle sprain trauma and not asymmetrical movement patterns as hypothesized. Rearfoot alignment, which could affect load patterns within the talocrural and STJ articulations, was not quantified in this sample. It is also important to note that this investigation is limited to T1ρ relaxation times and future research should evaluate the differences in cartilage volume and T2-mapping concurrently with T1ρ to elucidate the interactions among proteoglycan content, type-II collagen orientation, and morphology. Finally, the current design does not provide insights into how quickly, post index injury, these declines began or how such declines could be slowed via therapeutic interventions. Therefore, future longitudinal studies are needed to address these knowledge gaps.

## Conclusions

A CAI-involved limb has a higher T1ρ mean value (i.e., less proteoglycan content) at the talocrural and STJ than a CAI-contralateral and healthy control limb. Similarly, a CAI-involved limb has higher T1ρ variability at the talocrural joint relative to a healthy control but not the CAI-contralateral limb. A CAI-contralateral limb also had a higher T1ρ mean value (i.e., less proteoglycan content) at the posterior STJ relative to a healthy control limb. The talocrural and posterior STJ T1ρ means and variability have a strong positive association in a healthy control limb, but not in a CAI-involved limb. We conclude that CAI creates deleterious and MRI-measurable changes in ankle and subtalar joint cartilage composition that has implications for future models of clinical care, prevention, and research.
